# St. Bartholomew's Hospital

**Published:** 1919-10-25

**Authors:** 


### ST. BARTHOLOMEW'S HOSPITAL.

The foundation stone of St. Bartholomew's Hospital was laid in the year A.D. 1123, in the reign of Henry I. During nearly eight centuries of continuous effort in the cause of the sick and suffering, both the services rendered by this Hospital to the sick and to the medical profession, through its Medical School, have been of the utmost value. Dr. Addison, Minister of Health, in earnestly enforcing its irresistible claims to national and Metropolitan support, reminded his hearers that almost in every variety of public office, almost in every country, in every branch of national service, they would find men who had been trained at Bart's, a remarkable testimony in view of the eight successive centuries of splendid work done by St. Bartholomew's Hospital for the sick and suffering in all sections of the community throughout the Empire.

The Lord Mayor, Sir Horace Marshall, in his memorable year of office, has rendered notable services in many directions, and it is fitting that a closing act of his reign should be the wholehearted attempt which he is making that the City's own Hospital should be taken to heart by Londoners, whose privilege it is to see that this Institution's benevolent progress through the centuries shall not suffer a reverse. Sir Horace will be famous among Lord Mayors for the taste and attractiveness of his entertainments, and, if we may judge by the speeches of Dr. Addison, Lord Knutsford, Mr. Wilfrid Atlay, and other speakers, the luncheon at the Mansion House on Tuesday the 21st instant will prove a bumper success. Lord Knutsford, indeed, surpassed himself. He was brief, terse, humorous, and to the point. May we congratulate him upon the benefit he has evidently derived from his residence in Scotland, and the cheer which he enjoyed in being one to occupy the first train from Edinburgh to London, driven by volunteers, and the inspiriting experience brought to him by his commendable thoughtfulness in raising a sum from the passengers in testimony to their indebtedness and gratitude to the amateur drivers who brought them the 400 miles in safety without a hitch.

Mr. Wilfrid Atlay, Chairman of the Stock Exchange, in supporting the Lord Mayor, reminded Londoners that when he visited the Front during the War he found one hospital equipped and maintained entirely by a City institution, the Baltic, at a cost of £40,000 a year. Lord Sandhurst, in the course of his speech, made it perfectly clear that what St. Bartholomew's needs to-day is an additional income of £40,000 a year. Mr. Atlay eloquently claimed that what the Baltic could do in the War for one hospital the whole of London could immediately do for their own Hospital, St. Bartholomew's, which was making its first urgent appeal to the citizens, an appeal which no Londoner of Heart and Conscience could miss the opportunity and satisfaction of supporting. He was confident there would be a prompt and adequate response to the Lord Mayor's appeal for Bart's. In this connection it may be well to recall that the appeal made to raise an adequate sum to finance Guy's Hospital in its great distress and difficulties due to the fall in the value of land was completely successful in 1896. At a dinner at the Imperial Institute the equivalent in annual revenue and donations received after less than two months' continuous effort was upwards of £360,000. Subsequently the support grew, and that Prince of All Hospital Money-Raisers in the Present Century, Mr. Cosmo Bonsor, was so successful that the additions to the permanent finances of Guy's Hospital soon reached upwards of £1,000,000 sterling.

St. Bartholomew's Hospital is to-day in urgent need of at least £500,000. We should like to see our oldest Hospital triumphant. Following the Triumph of Guy's Hospital, the permanent resources of St. Bartholomew's should be augmented to the tune of £1,000,000 sterling before the Year of Victory is out. In any case, despite all the difficulties and abounding claims of 1919, in various directions, and the anxiety which weighs upon most people owing to the threatened heavy increases in taxation, we confidently believe that *Half a Million Sterling at least*, and possibly double that amount, may be banked to the credit of St. Bartholomew's Hospital before Easter 1920.

We cannot do better than republish in full the report of the proceedings at the Lord Mayor's banquet from *The Times* of the 22nd instant.

[From "The Times."]

A luncheon to open the People's Peace Year Commemoration Fund in aid of St. Bartholomew's Hospital was given at the Mansion House on the 21st inst., the Lord Mayor presiding.

Among those present were Lord Sandhurst (Treasurer) and Lady Sandhurst, Dr. Addison, Minister of Health, Lord Knutsford, Lord Aldenham, Sir Charles Wakefield, Sir John Bell, Sir Thomas MacKenzie, Sir Norman Moore (President, Royal College of Physicians), Sir George H. Makins (President, Royal College of Surgeons), Sir C. A. Hanson, M.P., Sir George Newman, Sir Henry C. Burdett, and Mr. E. Wilfrid Atlay (Chairman of the Stock Exchange).

The Lord Mayor read the following telegrams from the King and the Prince of Wales:

"I am desired to convey to your Lordship his Majesty's thanks for the loyal and dutiful message you have addressed to the King on the occasion of the luncheon you are giving at the Mansion House in support of the appeal that is being made on behalf of St. Bartholomew's Hospital, and in assuring you of the King's sympathy with the work of this great hospital I am but expressing the hope that your efforts to promote its interests may be attended with every success.—Cromer."

"I am desired by the Prince of Wales to express his regret at being unable to be present at the luncheon to-day in aid of St. Bartholomew's Hospital owing to his absence

**St. Bartholomew's Hospital**—(*continued*).

in Canada. As President of the Hospital, his Royal Highness hopes that the appeal will meet with every success.—Greville, Comptroller."

Proposing "Prosperity to St. Bartholomew's Hospital," **the Lord Mayor** said that since 1914 its annual cost of maintenance had risen by more than £30,000, while its income had actually fallen. The management was faced with a deficit of some £25,000, and if additional support was not obtained St. Bartholomew's would be compelled —he imagined for the first time in its 800 years of history —to reduce very materially its invaluable service to the community. This was the first time the management had appealed for funds for 150 years; and he trusted that under the happy auspices of its new President, the Prince of Wales, the hospital would enter on a new lease of life as a benefactor of the poor and afflicted.

**Dr. Addison** said this was not a mere casual appeal for funds for a well-established and useful institution. It was the appeal of an organisation which had rendered service to the sick and disabled continuously for hundreds of years—before St. Paul's Cathedral was built and before many of our Parliamentary institutions had their origin. The hospital was no longer an affair of the City of London, but of the nation. That was why he, as a member of the Government, and on their behalf, recommended his fellow-citizens to support this fund. As a student of the hospital himself, he knew that there was, and had been, in St. Bartholomew's a singularly high standard of exactness and duty in its services for many generations, and also a standard of courtesy and consideration to the poorest patient. The authorities of the hospital had decided to provide better accommodation for nurses, and the Government welcomed that decision. The Government grant in aid of the organisation and support of a clinical unit would be first claimed by this hospital. It had been the first in the country to establish a medical and surgical clinical union, and as it had put itself in the van in this matter, he felt that it was deserving of public support. In every branch of national service they would find men who had been trained in the traditions of St. Bartholomew's Hospital, and, believing that it had a great claim on the support of the nation, he supported the appeal.

**Lord Sandhurst,** replying, explained that the circumstances that had led to this appeal were the rise in prices in every direction. Their wages bill since 1914 had increased to close on £20,000 a year. St. Bartholomew's was now in need, but they might rely upon it no stone would be left unturned to get the funds required. They had made a start that day in the great *Times* newspaper; and they wanted the appeal to be as widespread as possible and to permeate every possible class.

**Lord Knutsford, Mr. E. Wilfrid Atlay, and Sir Norman Moore** strongly supported the appeal, and at the close of the proceedings the Lord Mayor announced that the Mansion House appeal had resulted in an inaugural fund of £18,000.

The first list of subscribers of £1,000 are: London County and Westminster Bank, Limited; Barclay's Bank, Limited; Lloyds Bank, Limited; London Joint City and Midland Bank, Limited; Anglo-South American Bank, Limited; Mr. F. C. Stoop, the National Provincial and Union Bank of England, Limited; and Sir Oswald Stoll.

